# Reshaping of soil carbon and nitrogen contents in quincentenary ancient rice terraces: The role of both short-term abandonment and prokaryotic functional groups

**DOI:** 10.3389/fmicb.2022.1007237

**Published:** 2022-12-01

**Authors:** Wenqing Li, Zhongwu Li, Yaojun Liu, Xiaodong Nie, Chuxiong Deng, Guangye Zhang, Shuyuan Wang, Tao Xiao, Han Zheng

**Affiliations:** ^1^School of Geographic Sciences, Hunan Normal University, Changsha, China; ^2^Key Laboratory of Subtropical Ecology and Environmental Change, Hunan Normal University, Changsha, China

**Keywords:** ancient rice terraces abandonment, drylands and forestlands, soil profile, soil carbon and nitrogen contents, soil prokaryotic communities, functional groups

## Abstract

Microbial communities and functions play an important role in soil carbon and nitrogen transformations, and, in recent decades, the abandonment of terraces is prevalant in the hilly areas of China. However, it is unclear how soil carbon and nitrogen contents and prokaryotic communities changed as a result of the abandonment of ancient rice terraces. Soil profiles ranging from 0 to 120 cm were excavated on drylands, forestlands (both converted due to the abandonment of ancient rice terraces), and ancient rice terraces. The FAPROTAX database was used to predict soil prokaryotic functional groups. The results showed that soil organic carbon (SOC) and total nitrogen (TN) contents of abandoned ancient rice terraces in drylands (51.09 and 33.20%) and forestlands (31.76 and 16.59%) were significantly reduced. Soil prokaryotic diversity and community composition changed dramatically after the abandonment of terraces and were mainly affected by soil pH and ammoniacal nitrogen (${\text{NH}}_{4}^{+}$-N). Community composition was more similar in drylands and forestlands. Moreover, the abundance of transformation functional genes of carbon (57.01 and 50.80%) and nitrogen (15.25 and 22.36%) in bacterial communities was significantly reduced, and of carbon in the archaeal communities decreased sharply (28.10 and 46.50%), in drylands and forestlands. These findings indicate that short-term abandonment of ancient rice terraces reduces soil carbon and nitrogen contents, which may be closely related to the decline of prokaryotic functional groups. The prevalence of short-term abandonment of rice terraces in the hilly areas of China may pose adverse ecological risks.

## Introduction

In the hilly areas of China, most of the land is not particularly suitable for agricultural production because reclamation on steep slopes can cause ecological problems such as soil erosion and nutrient loss (Deng et al., 2021; Li W. et al., 2022). As an important engineering measure for soil and water conservation, terraces can effectively alleviate these problems and are widely promoted (Deng et al., 2021). Terraces account for 26.43% of China's cropland area, which has effectively guaranteed the basic needs of agricultural food production from ancient times to the present (Lansing et al., 2017; Cao et al., 2021). However, factors, such as low agricultural labor benefits, scattered farming operations, and complicated mechanization, have caused a large number of rice terraces to be transformed into woodlands, drylands, and even wastelands (Arnaez et al., 2011; Zhang et al., 2019). Such abandonment of agricultural terraces is also prevalent in other countries or regions (Stavi et al., 2019; Djuma et al., 2020). There have been positive or negative results on the effect of the abandonment of terraces on soil carbon and nitrogen contents (Djuma et al., 2020; Li W. et al., 2022), and the reason for this controversy may be the neglect of an important role of soil microorganisms in it. Soil microorganisms play an important role in material cycling and nutrient transformation, affecting soil carbon and nitrogen contents (Falkowski et al., 2008; Louca et al., 2016). To date, understanding the effects of abandonment of rice terraces on soil carbon and nitrogen contents has relied more on evidence from soil aggregates and erosion (Arnaez et al., 2011; Li W. et al., 2022), while the biological information from short-term abandonment of ancient rice terrace on the fate of soil microorganisms remains ambiguous.

Soil prokaryotic communities (bacteria and archaea) have extremely high species and genetic diversity as well as extensive niches in terrestrial ecosystems, which are closely related to energy flow and material cycling (Fierer and Jackson, 2006; Falkowski et al., 2008). As important drivers and participants in the biogeochemical cycles of ecosystems, soil prokaryotes can influence numerous ecological processes through catabolism and anabolism (Falkowski et al., 2008; Li W.-Q. et al., 2022). For example, carbon and nitrogen cycles are affected by changing the composition and mineralization rate of soil organic matter. Moreover, prokaryotes with different potential ecological functional units can directly affect the soil carbon and nitrogen cycle, changing the quantity of their contents and affecting turnover (Louca et al., 2016). Gao et al. (2020) found that, after grassland and farmland soil organic carbon (SOC) increased significantly, the abundance of related prokaryotic community functional genes also increased sharply, and similar results were also observed in a study by Lu et al. (2017). Therefore, functional groups related to carbon and nitrogen transformations in the prokaryotic communities can better reflect soil carbon and nitrogen. The FAPROTAX database, which is skilled and widely used, can predict soil carbon and nitrogen-related transformation in prokaryotic functional group genes (Yang et al., 2022). In fact, the community composition and function of prokaryotes can alter soil carbon and nitrogen contents, but it is still unclear how they respond to the abandonment of ancient rice terraces.

The abandonment of terraces can destroy their structure and function, and the corresponding engineering effects are lost, which in turn significantly change soil properties (Stavi et al., 2019). The natural abandonment process of rice terraces exerts critical abiotic pressures (nutrient or pH variability) on soil microbial community composition and functional group diversity (Camenzind et al., 2018). Gubry-Rangin et al. (2017) found that pH significantly affected archaeal physiology, including reproduction in the soil environment and optimum active temperature. Kutvonen et al. (2015) showed that, when soil nitrogen compounds were available in sufficient amounts, bacterial abundance increased significantly and specific taxonomic units were enriched. Previous studies found that the abandonment of terraces can change soil nutrients and texture (Ramos et al., 2007; Xiao et al., 2018). Prokaryotic community composition and functional groups may be altered by the abandonment of terraces because their soil properties are distinguishable. Meanwhile, assessing the impact of soil property changes caused by the abandonment of ancient rice terraces on the prokaryotic communities should be explored.

Studies on the variability of prokaryotic community composition and functional groups have been more biased toward surface soils, but little is known about its response in the deep layer (Eilers et al., 2012; Yuan et al., 2020). Ancient rice terraces have experienced hundreds of years of management measures (repeated tillage and fertilization), as well as long-term leaching and enrichment effects, resulting in significant soil horizons in the profile (Gong, 1995). The environment varies significantly in different soil horizons, and the prokaryotic community adopts appropriate strategies to adapt to the environment, which will affect soil carbon and nitrogen transformation processes (Li W.-Q. et al., 2022; Yang et al., 2022). Meanwhile, clarifying the changes in the prokaryotic community structure and function in different soil horizons can provide a better understanding of the ecological consequences of the abandonment of ancient rice terraces.

In recent decades, the abandonment phenomenon of rice terraces has become popular in the hilly areas of China (Zhang et al., 2019; Deng et al., 2021), which has led to a series of problems such as soil erosion, nutrient loss, and low moisture (Arnaez et al., 2011; Stavi et al., 2019). Previous studies focused more on soil erosion, nutrient content, and enzyme activity (Xiao et al., 2018; Stavi et al., 2019). Fewer studies focused on the response of the prokaryotic communities and functions to the abandonment of rice terraces, and it is also unclear how it affects soil carbon and nitrogen contents. This study hypothesized that the abandonment of ancient rice terraces would reduce contents, which might be closely related to changes in prokaryotic community composition and functions. The objectives of this study were (1) to explore the effects of the abandonment of ancient rice terraces on contents; (2) to identify the factors influencing soil prokaryotic communities; and (3) to elucidate the possible associations of the prokaryotic communities with contents.

## Materials and methods

### Experimental area

**Figure 1:**
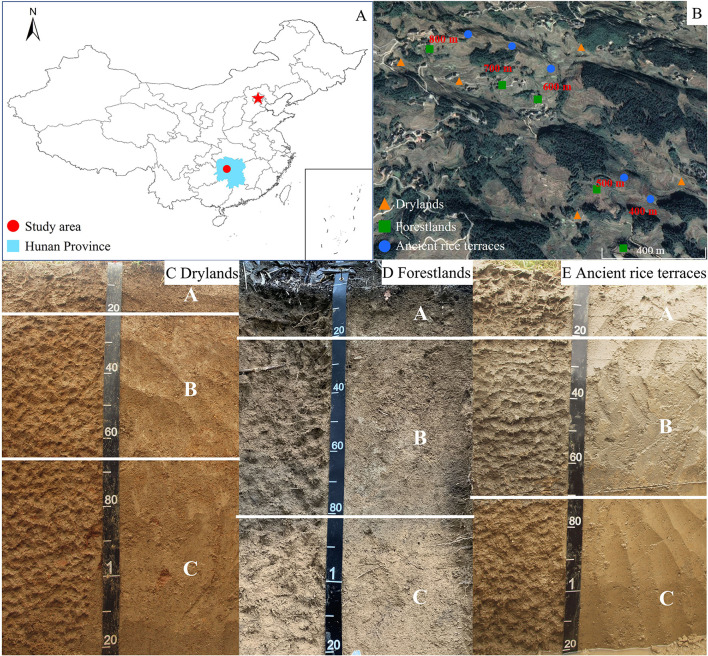
The geographical location **(A)**, sample site distribution **(B)**, and soil profile **(C–E)** of the experimental plot.

A typical representative of rice terraces in the hilly areas of China is Ziquejie ancient rice terraces (Xinhua County, Hunan province, 27°30′-27°45′N, 110°52′-111°21′E; Figure 1) (Li et al., 2020). The area experiences simultaneous rain and heat in a humid subtropical monsoon climate. The average annual temperature and precipitations are 16°C and 1,650–1,700 mm, respectively. The altitude ranges from 400 to 1,500 m. Red earth soils (Ultisols) developed from granite and its weathered debris has a good structure and permeability.

After hundreds of years of agricultural development, Ziquejie has formed a unique rice terrace landscape and is named “Global Important Agricultural Cultural Heritage” in 2018 by the United Nations Food and Agriculture Organization. For a long period of time, rice cultivation has been the primary grain production and economic income of local residents. However, terraces have poor farming conditions (small area and wide distribution) and the benefit of low labor input. These adverse factors broke the traditional small-scale peasant economy with young labor force moving to the city to obtain higher returns, resulting in the abandonment of more and more terraces. Ancient rice terraces mainly form drylands and forestlands after the abandonment. Sweet potato (*Ipomoea batatas*) is mainly planted on drylands, and Chinese fir (*Cunninghamia lanceolata*) is mainly planted on forestlands. In recent decades, the abandonment phenomenon of agricultural terraces has been popular in the hilly areas of China (Zhang et al., 2019).

Three soil samples taken from different soil depths in Ziquejie ancient rice terraces were sent to the Accelerator Mass Spectrometry Laboratory at the Beta Analytic Testing Laboratory for AMS ^14^C dating, and it was found that the probability of 549–608-years-old in the 0–20 cm soil layer was 95.4% (Supplementary Table S1). The lower soil also had a long calendar age.

### Field sampling and chemical determination

Ancient rice terraces, drylands, and forestlands have relatively intact continuous slopes and similar topographic and geomorphological conditions. Drylands and forestlands were formed by the abandonment of ancient rice terraces for 15~20 years, with the same initial soil properties. Repeated tillage, dry-wet alternation, fertilizer application, and long-term leaching and enrichment utility in rice terraces result in significant soil horizons (Figure 1). After more than 10 years of abandonment, soil horizons can still be seen clearly in three land use types. According to the characteristics and horizon of soil development (Gong, 1995), 0–20 cm is divided into surface soil (A), 20–70 cm is divided into subsoil soil (B), and 70–120 cm is divided into substratum (C). Soil samples of three land use types were collected at each altitude (altitudes: 400, 500, 600, 700, and 800 m), with an interval of 80–200 m along the contour between the different land use types at the same altitude (Figure 1). Three soil profiles were randomly taken, and soil samples from the different soil layers were mixed evenly in a 20 m × 20 m sample square in the same land use type and altitude. Of the soil profile from 0 to 120 cm, one sample was collected between 0 and 20 cm, two samples were collected between 20 and 70 cm, and three samples were collected between 70 and 120 cm. All soil samples were collected at the same time. Pebbles and plant residues were removed from the soil and mixed thoroughly, passed through a 2-mm sieve, and then quickly transported back to the laboratory. The transported mixture was stored at −80°C for Illumina MiSeq high-throughput sequencing, held at 4°C for determining available nutrients, and air-dried for determining total nutrients.

In air-dried soils, soil pH was determined using a pH meter (v/w, water/soil = 2.5/1) (Metter-Toledo, Switzerland), SOC by K_2_Cr_2_O_7_ volumetric dilution heating method, TN using an elemental analyzer (Elementar, Germany), total phosphorus (TP) by the alkaline NaOH fusion–molybdenum antimony colorimetric method, and cation exchange capacity (CEC) by the ammonium acetate extraction process. In fresh soils, alkaline nitrogen (AN) was determined by UV-1601 spectrophotometry (Shimadzu, Japan), available phosphorus (AP) by molybdate-ascorbic acid method, nitrate nitrogen (${\text{NO}}_{3}^{-}$-N) and ${\text{NH}}_{4}^{+}$-N by indophenol blue spectrophotometric method using UV-1601 spectrophotometry (Shimadzu, Japan). The measured physicochemical properties of soil are shown in Supplementary Table S2.

### Bioinformatic analyses

The FastDNA^®^ Spin kit for Soil (MP Biomedicals, USA) was used for the extraction of deoxyribonucleic acid (DNA) according to the manufacturer's instructions. V4 and V4V5 of the 16S ribosomal ribonucleic acid (rRNA) hypervariable region of bacterial and archaeal communities were amplified by primer pairs. Primer sets of 338F (AYTGGGYDTAAAGNG)−806R (TACNVGGGTATCTAATCC) and 340F157 (TGYCAGCCGCCGCGGTAA)−1000F (YCCGGCGTTGAVTCCAATT) were used to amplify the target fragment, respectively (Caporaso et al., 2012). Polymerase chain reactions (PCRs) were performed according to the following procedure: denaturation at 95°C for 3 min; 27 cycles of 95°C for 30 s, annealing at 55°C for 30 s, extension at 72°C for 45 s, and a final extension at 72°C for 10 min. PCRs were repeated three times in a mixture containing 0.4 μl of FastPfu Polymerase, 0.8 μl of each primer (5 μM), 2 μl of 2.5 mM deoxynucleoside triphosphates (dNTPs), 4 μl of 5 × FastPfu Buffer, and 10 ng of template DNA for a total of 20 μl. Then, the PCRs were purified using the AxyPrep DNA Gel Extraction kit (Axygen Biosciences, USA) and further quantified using QuantiFluor™-ST (Promega, USA). Finally, the purified amplification products were combined in equimolar ratios and sequenced on the Illumina Miseq platform at Shanghai Personal Biotechnology, Co., Ltd., China.

Raw reads generated by Illumina Miseq sequencing were trimmed and quality filtered using Quantitative Insights into Microbial Ecology, v1.8.0 (QIIME1). UPARSE (version 7.1 http://drive5.com/uparse/) with a novel “greedy” algorithm clustered operational taxonomic units with a similarity cut-off of 97% and performed both chimera filtering and operational taxonomic unit clustering (Edgar, 2013). For taxonomic annotation, bacteria and archaea were classified using the Ribosomal Database Project classifier (v2.2) against the database for each 16S rRNA gene sequence (Pruesse et al., 2007). The classification of bacteria and archaea was performed using the Ribosomal Database Project Bayesian classifier, and their relative abundance was calculated by calculating the number of sequences and ribosomal DNA (rDNA) gene sequences detected in the sample. Diversity and richness were calculated by Chao 1 and Shannon through the Mothur program (Schloss et al., 2009).

### Data analyses

Differences in soil properties and prokaryotic communities in different soil horizons and land use types were determined using a one-way analysis of variance (ANOVA), and the effects of soil horizons and land use types were analyzed by two-way ANOVA. A statistically significant difference was based on the 0.05 level using SPSS 22.0 software (IBM, USA). Nonmetric multidimensional scaling was used to analyze the variation in the prokaryotic community structure among land use types in different soil horizons by Bray–Curtis distances using the Multiple Response Permutation Procedure in R (V 3.5.0), and a similarity analysis was used to perform an ANOVA between groups based on Bray–Curtis distances. To determine the influence of soil properties on prokaryotic community composition, a redundancy analysis was performed using Canoco 5.0 software (Microcomputer Power, USA) to identify significant influencing factors using the Monte Carlo permutation test (499 permutations, *p* < 0.05). To explore the relationship between the prokaryotic communities and soil properties, Pearson's correlation coefficients were calculated using Origin 2022 (OriginLab, USA). The prokaryotic taxa (FAPROTAX) database was used to predict functional groups based on 16S rRNA genes (Louca et al., 2016).

## Results

### Soil carbon and nitrogen contents

**Figure 2:**
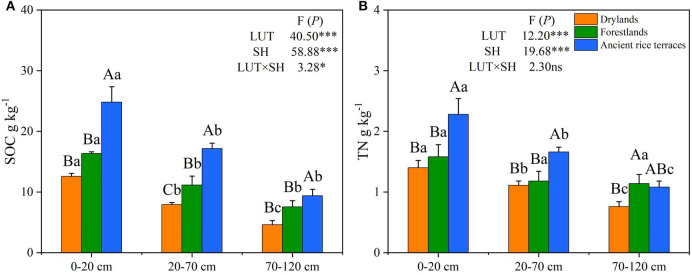
**(A,B)** SOC and TN contents of three land use types in different soil horizons. SOC, soil organic carbon; TN, total nitrogen; LUT, land use types; SH, soil horizons. Various capital letters stand for significant differences among diverse land use types, diverse lower-case letters indicate significant differences among diverse soil horizons. *p < 0.05; ***p < 0.001; ^ns^ not significant.

The abandonment of ancient rice terraces to drylands and forestlands significantly declined SOC and TN contents and changed their vertical distribution (Figure 2). Compared with ancient rice terraces, SOC content in drylands and forestlands was considerably decreased by 51.09 and 31.76%, respectively. SOC content was significantly higher in the 0–20-cm soil horizon than in the 20–70- and 70–120-cm soil horizons. TN content was significantly lower in drylands and forestlands than in ancient rice terraces by 33.20 and 16.59%, respectively. The vertical distribution of TN showed a similar variation to that of SOC. Land use types and soil horizons had a significant effect on SOC and TN contents (*p* < 0.001).

### Soil prokaryotic diversity and community composition

**Figure 3:**
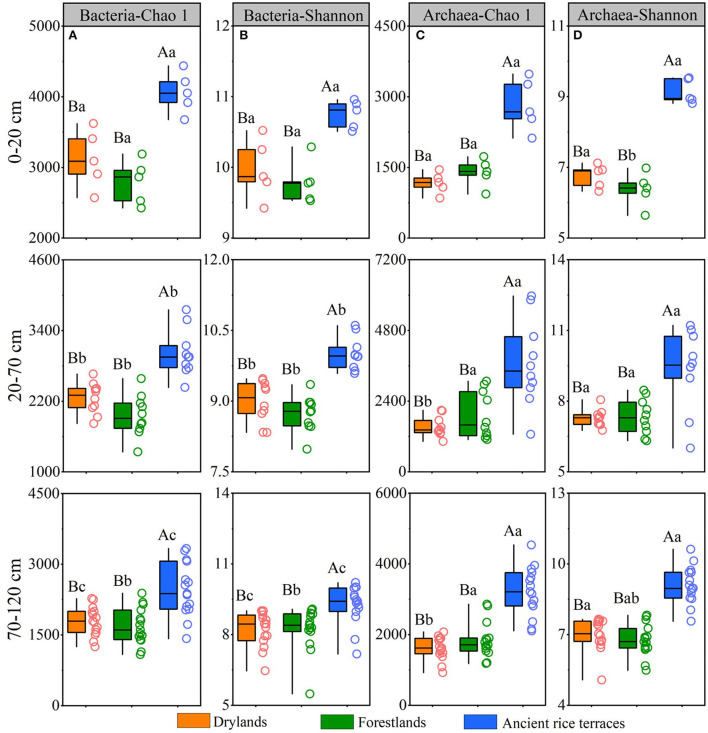
Soil prokaryotic richness and diversity of three land use types in different soil horizons. Various capital letters stand for significant differences among diverse land use types, and diverse lower-case letters indicate significant differences among diverse soil horizons.

A total of 8,372,676 and 4,645,899 reads were obtained from all soil samples for bacteria and archaea, respectively (Supplementary Table S3). The abandonment of ancient rice terraces significantly reduced the richness and diversity of prokaryotes in different soil horizons, and there was an apparent downward trend of soil bacteria in ancient rice terraces with a soil profile variation (Figure 3).

**Figure 4:**
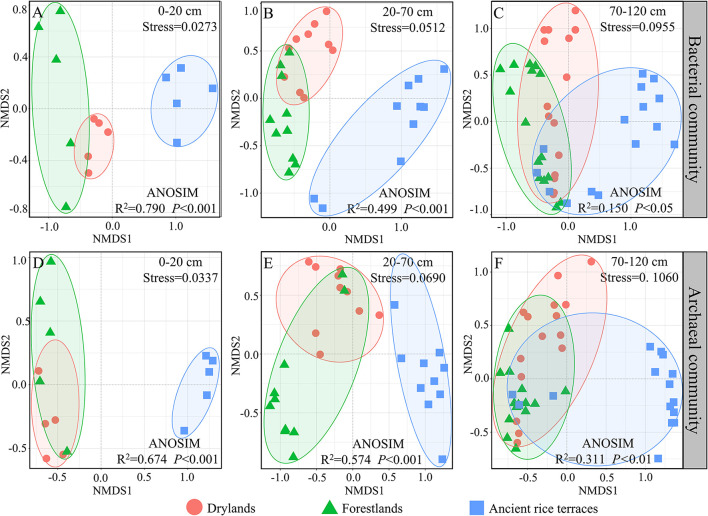
**(A–F)** A nonmetric multidimensional scaling analysis of soil prokaryotic community composition of three land use types in different soil horizons based on Bray–Curtis dissimilarities. Analysis of similarity with the Bray–Curtis distance to test whether sample distributions are significantly different.

Nonmetric multidimensional scaling showed that bacterial and archaeal communities in drylands and forestlands always intersected and separated from ancient rice terraces in the 0–20- and 20–70-cm soil horizons; eventually, they would intersect in the 70–120-cm soil horizon (Figure 4).

*Proteobacteria* and *Acidobacteria* accounted for 26.80 and 27.18%, respectively, and were the main bacterial phyla (Supplementary Figure S1). In different soil horizons, the relative abundance of *Proteobacteria* was enormously higher in ancient rice terraces than in drylands and forestlands but remarkably decreased with varying soil profile. *Acidobacteria* and *Chloroflexib* had the highest relative abundance in drylands and forestlands. *Thaumarchaeota* and *Euryarchaeota* accounted for 42.26 and 30.95%, respectively, of relative abundance in drylands and forestlands and were the main archaeal phyla. In different soil horizons, the relative abundance of *Thaumarchaeota* was remarkably lower in ancient rice terraces than in drylands and forestlands, but the opposite was true for *Crenarchaeota* and *Asgardaeota*.

### Predicted potential function of soil prokaryotic communities

**Figure 5:**
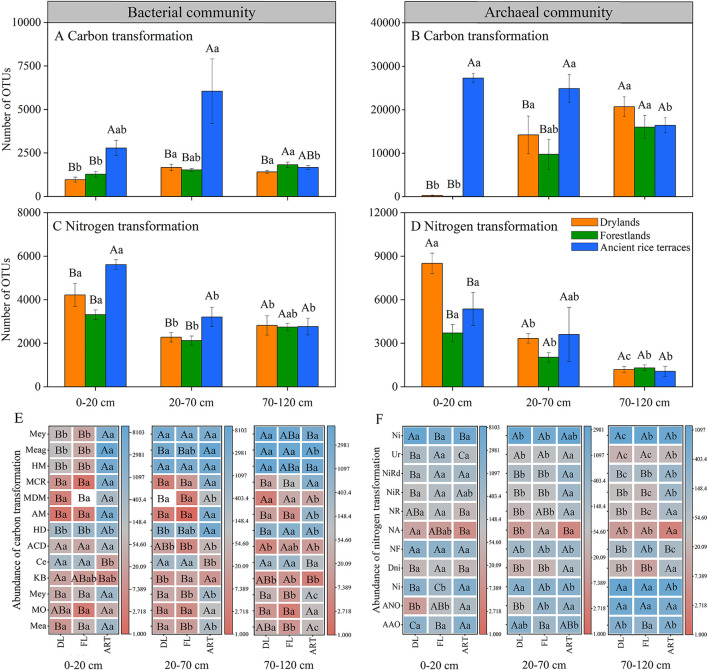
Prokaryotic functional annotation (FAPROTAX) analysis was used to evaluate the abundance of carbon and nitrogen transformations in soil prokaryotic communities of three land use types in different soil horizons. Various capital letters stand for significant differences among diverse land use types, diverse lower-case letters indicate significant differences among diverse soil horizons. **(A–D)** Indicate the abundance of total carbon and nitrogen transformations in soil bacterial and archaeal communities, respectively; **(E,F)** indicate the abundance of individually transformed genes. Carbon transformation (bacterial communities: Mea, methanotrophy; MO, methanol oxidation; Mey, methylotrophy; KB, knallgas bacteria; Ce, cellulolysis; ACD, aromatic compound degradation; HD, hydrocarbon degradation. Archaeal communities: AM, acetoclastic_methanogenesis; MDM, methanogenesis by disproportionation of methyl groups; MCR, methanogenesis by CO_2_ reduction with H_2_; HM, hydrogenotrophic methanogenesis; Meag, methanogenesis; Mey, methylotrophy). Nitrogen transformation (bacterial communities: AAO, aerobic ammonia oxidation; ANO, aerobic nitrite oxidation; Ni, nitrification; Dni, denitrification; NF, nitrogen fixation; NA, nitrate ammonification; NR, nitrite respiration; NiR, nitrate respiration; NiRd, nitrate reduction; Ur, ureolysis. Archaeal communities: Ni, nitrification).

The FAPROTAX database was used to predict carbon and nitrogen cycle-related prokaryotic functional groups. The abundance of carbon and nitrogen transformation genes in the soil bacterial community were significantly decreased in the 0–20- and 20–70-cm soil horizons after the abandonment of ancient rice terraces, and functional group methanotrophy, methanol oxidation, methylotrophy, and hydrocarbon degradation showed similar trends (Figure 5). In drylands andforestlands, soil archaeal community carbon transformation was significantly reduced in the 0–20- and 20–70-cm soil horizons.

### Relationship between soil carbon and nitrogen and prokaryotic communities

**Figure 6:**
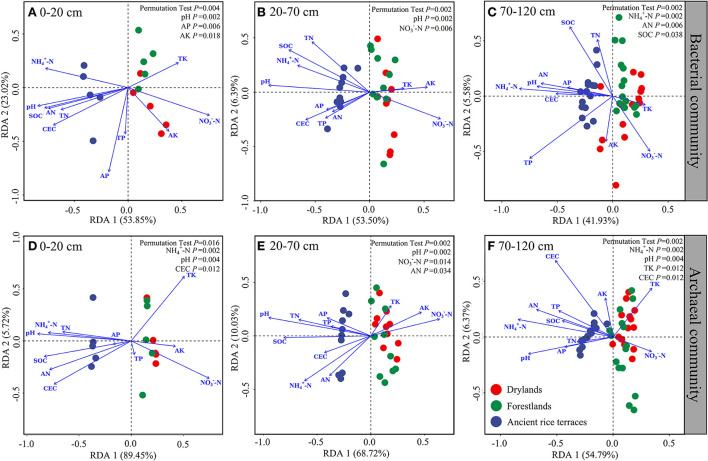
**(A–F)** Redundancy analysis of soil properties and prokaryotic communities in different soil horizons.

The full explanatory degrees of bacterial community changes were 76.87% (*p* = 0.004), 59.89% (*p* = 0.002), and 47.51% (*p* = 0.002), respectively, for the 0–20, 20–70, and 70–120 cm soil horizons (Figure 6). Monte Carlo permutation tests showed significant effects in the 0–20-cm soil horizon for pH, AP, and AK; 20–70-cm soil horizon for pH and ${\text{NO}}_{3}^{-}$-N; and 70–120-cm soil horizon for ${\text{NH}}_{4}^{+}$-N, AN, and SOC. The total explanatory degrees of variation in the archaeal communities were 95.17% (*p* = 0.016), 78.30% (*p* = 0.002), and 61.15% (*p* = 0.002), respectively, for the 0–20, 20–70, and 70–120 cm soil horizons. Monte Carlo permutation tests showed notable effects in the 0–20-cm soil horizon for ${\text{NH}}_{4}^{+}$-N, pH, and CEC; 20–70-cm soil horizon for pH, ${\text{NO}}_{3}^{-}$-N, and AN; and 70–120-cm soil horizon for ${\text{NH}}_{4}^{+}$-N, pH, TK, and CEC.

**Figure 7:**
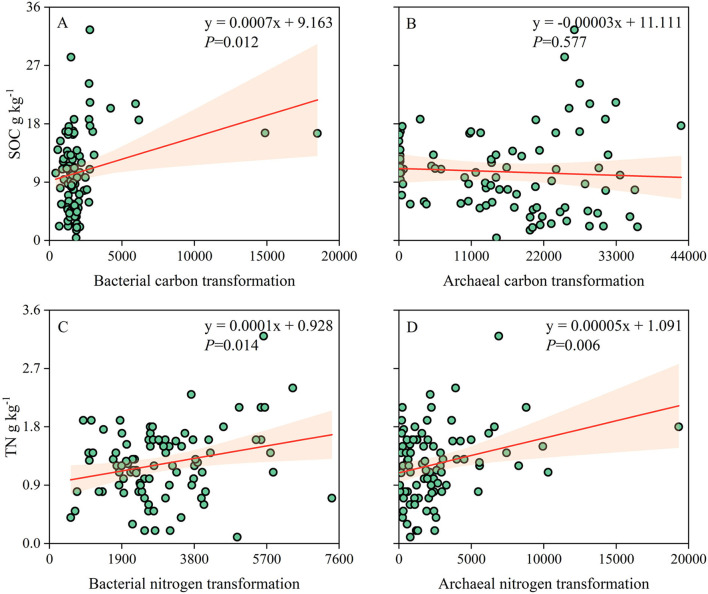
**(A–D)** Relationships of soil prokaryotic community carbon and nitrogen transformations with SOC and TN contents.

The correlation analysis showed that carbon and nitrogen transformations of the soil bacterial community were significantly and positively correlated with SOC (*p* = 0.012) and TN (*p* = 0.014). Nitrogen transformation of the archaeal community was positively correlated with TN (*p* = 0.006), while carbon transformation was not significantly correlated with SOC (*p* = 0.577; Figure 7). SOC and TN contents were highly associated and consistent with carbon- and nitrogen-related functional groups (Supplementary Figure S2).

## Discussion

### Effects of the abandonment of ancient rice terraces on soil carbon and nitrogen contents

The abandonment of ancient rice terraces reduced soil carbon and nitrogen contents, as potentially expected. Xiao et al. (2018) found that there was a significant decline in SOC and TN contents after the abandonment of terraces in the hilly and gully regions. A meta-analysis by Chen et al. (2020) found that terraces can increase SOC sequestration by 32.4% from a nationwide data analysis in China. In addition, carbon and nitrogen are frequently coupled, which also indicate that terraces are favorable for increasing soil nitrogen contents (Deng et al., 2021). There are several possible reasons for the reduction of SOC and TN contents after the abandonment of ancient rice terraces. First, there is a difference in nutrient sources. Terraces have a wide range of nutrient sources such as stalks, weeds, and other plant residues and fertilizers. Ancient rice terraces are distributed close to villages, and it is easy to apply farmyard manure and raise ducks and fish (Supplementary Figure S3), which can effectively improve soil carbon and nitrogen contents (Li W. et al., 2022). However, drylands and forestlands tend to be more distant and less accessible, less disturbed by human and livestock activities, and have fewer external nutrient inputs. Second, there is the presence or absence of terrace management. Terrace is a vital soil and water conservation engineering measure, which has many essential functions such as maintaining nutrients and reducing soil erosion (Deng et al., 2021). The abandonment of rice terraces reduces the original function. After the collapse of terrace walls and ridges, soil is more vulnerable to rain runoff erosion and leaching, resulting in the loss of soil carbon, nitrogen, and other nutrients (Deng et al., 2021; Li X.-Z. et al., 2022). Third, whether the soil is submerged. Long-term flooding of rice terraces increases the content of elements, such as iron and manganese, which is conducive to the stability and protection of soil organic matter (Wang et al., 2017). Additionally, the flooding of rice terraces inhibits microbial activity and reduces the mineralization of soil organic matter, which promotes the retention of refractory components in plant residues (Chen et al., 2021). Overall, terraces are favorable for the accumulation of soil carbon and nitrogen contents due to their own characteristics and human activities (Chen et al., 2020; Deng et al., 2021). When terraces are abandoned, a vital ecosystem service function becomes weaker, and an ecological impact is manifested in the reduction of soil carbon and nitrogen contents.

Soil organic carbon and TN contents decreased with the soil horizon, which was consistent with the findings of Yuan et al. (2020) and Li W. et al. (2022). In this study, SOC and TN contents were higher in the 0–20-cm soil horizon than in the 20–120- and 70–120-cm soil horizons. Tillage can prevent rice roots from spreading into the deep layer and indirectly enables the surface layer to obtain more root carbon sources and secretions than the bottom layer (Chen et al., 2021). Although the roots of drylands and forestlands can break through the plow pan, the law of soil carbon and nitrogen contents decreasing with the increase of soil depth is still applicable. On the one hand, plant litter is the first to decompose in the surface layer, which can accumulate a large amount of plant carbon and nitrogen that is easily accessible to the soil (Li W. et al., 2022). Although soil nutrients can enter the substratum through leaching and infiltration, they are at a low level. In addition, the surface soil has finer roots and root secretions, which can promote the accumulation of soil carbon and nitrogen contents (Li et al., 2021; Li W. et al., 2022).

### Effects of the abandonment of ancient rice terraces on soil prokaryotic communities

Although the relative abundance and composition of bacterial communities differed significantly among the three land use types, they shared a similar microbial taxon, which could be because they all originated from ancient rice terraces (Chen et al., 2022). In this study, *Proteobacteria* decreased after the abandonment of ancient rice terraces, while *Acidobacteria* and *Chloroflexib* were elevated due to their different life history strategies. As r-strategists, *Proteobacteria* are adapted to growing in nutrient-rich soils and can achieve intrinsic growth potential at the fastest rate (Fierer et al., 2007; Li W.-Q. et al., 2022). *Acidobacteria* and *Chloroflexib* are K-strategists that can degrade lignin and cellulose to grow in a low-carbon environment due to their good affinity with substrates (Fierer et al., 2007; Li W.-Q. et al., 2022). Thus, differences in soil nutrients caused by the abandonment of ancient rice terraces altered the relative abundance of bacterial communities. Previous studies showed that *Acidobacteria* were highly correlated with soil pH in the range of 3.5–6.0, and their relative abundance was higher in acidic soils (Fierer and Jackson, 2006). In this study, drylands and forestlands have a relatively acidic environment, which favors the reproduction of *Acidobacteria*. Rice terraces are prone to redox reactions due to prolonged flooding, and it is easy to raise soil pH value through the decarboxylation of organic acid anions (Ding et al., 2019).

The relative abundance and composition of archaeal communities differed significantly after the abandonment of ancient rice terraces, which might be similar to the reasons for bacterial community changes. In this study, *Thaumarchaeota* was significantly higher in drylands and forestlands than in ancient rice terraces, while *Euryarchaeota* showed the opposite. *Thaumarchaeota* is an oxygen-consuming archaeal taxon, and Gubry-Rangin et al. (2017) found that ammonia-oxidizing archaea were negatively correlated with pH using a polynomial nonlinear relationship to fit. The sealing and pH value of ancient rice terraces are higher than those of drylands and forestlands, resulting in a relatively low abundance of *Thaumarchaeota* in the terraces, only 4.95%. Hu et al. (2013) found that the relative abundance of *Euryarchaeota* showed a highly significant linear relevance to soil moisture and ${\text{NH}}_{4}^{+}$-N, and soil moisture above 20% favors its growth. Thus, ancient rice terraces have a more suitable living environment. Furthermore, the particular membrane function of *Euryachaeota* can reduce unnecessary ion circulation to minimize energy loss, which makes it more adaptable to flooded environments (Hu et al., 2013).

Paddy soil is formed through maturation under the influence of human activities, such as periodic irrigation and drainage, which is completely different from dryland soils (Novair et al., 2020). Long-term redox in rice terraces is more likely to promote the accumulation of carbon and nutrients in the topsoil, which favors the growth of r-strategic microbes (Fierer et al., 2007; Chen et al., 2021). The study area is located in a typical red soil hilly region, which is rich in iron and aluminum oxides. Thus, the conversion of ancient rice terraces to drylands and forestlands reduced the pH value due to the oxidation of large amounts of iron, which increased the relative abundance of *Acidobacteria* (Fierer and Jackson, 2006). Based on differences in oxidation or reduction conditions between paddy and dryland soils, this resulted in a more similar prokaryotic community structure between drylands and forestlands in the surface layer, while differing significantly from ancient rice terraces. In the substratum, water does not penetrate smoothly, weakening the differences caused by redox (Yuan et al., 2020), which results in a closer prokaryotic community for the three land use types, a result proven by nonmetric multidimensional scaling.

A redundancy analysis showed that soil pH and ${\text{NH}}_{4}^{+}$-N has an important influence on driving the variability of prokaryotic community composition. Soil pH can affect the chemical form, concentration, and availability of substrates and acts directly on microorganisms to screen out an adaptable taxon (Nicol et al., 2008). ${\text{NH}}_{4}^{+}$-N is a critical source of available nitrogen for soil microorganisms and a cardinal limiting factor for microbial proliferation (Kutvonen et al., 2015). In this study, ${\text{NH}}_{4}^{+}$-N was not affected by soil horizons, but pH was affected. Among the three land use types, the pH value did not change significantly in the soil profile of ancient rice terraces while drylands and forestlands increased significantly with soil depth. This may also account for the closer soil prokaryotic community composition in drylands and forestlands.

### Changes in the functional groups of soil prokaryotic carbon and nitrogen transformations

Soil microorganisms have an irreplaceable role in the carbon and nitrogen cycle of agroforestry ecosystems and are a highly active part of the biosphere (Falkowski et al., 2008; Monteux et al., 2020). Understanding the altered functional group characteristics of soil microbial communities can better elucidate the ecological effects of the abandonment of ancient rice terraces on soil carbon and nitrogen. Several studies showed that the transformation functional group gene abundance of soil microbial carbon and nitrogen has a strong correlation with the carbon and nitrogen cycle, which can significantly affect their turnover and status (Gao et al., 2020; Jiang et al., 2021). In this study, the abundance of transformation genes of soil prokaryotic carbon and nitrogen was significantly reduced after the abandonment of ancient rice terraces, except for archaeal nitrogen transformation, and SOC and TN contents showed the same reduction trend. While transformation genes of bacterial carbon and nitrogen were significantly associated with SOC and TN contents, transformation genes of only archaeal nitrogen were significantly associated with TN content. Wang et al. (2021) reported that soil bacterial genes contributed 84.53 and 95.62% to carbon decomposition and fixation, respectively, while archaeal genes contributed only 1.09 and 4.05%, respectively, through the detection of functional genes in soils of desert ecosystem. It may be that archaea are more biased toward soil nitrogen transformation, while bacteria specialize in carbon and nitrogen transformations (Monteux et al., 2020; Wang et al., 2021). Collectively, the abandonment of ancient rice terraces changed soil properties, such as pH and ${\text{NH}}_{4}^{+}$-N, which in turn altered the bacterial and archaeal community structures and reduced the gene abundance of the transformationfunctional groups of carbon and nitrogen. This caused a decrease in soil carbon and nitrogen contents. Although previous studies found that the abandonment of ancient rice terraces increased soil carbon and nitrogen contents, this was over a long-time scale or specific vegetation types (Ramos et al., 2007; Djuma et al., 2020; Li W. et al., 2022). However, our study focused more on the effects of short-term abandonment of rice terraces, which should be taken into account.

Taken together, we speculated that soil bacterial communities shifted from r- to K-strategists and that soil archaeal communities changed from *Euryarchaeota* to *Thaumarchaeota* after the conversion of ancient rice terraces to drylands and forestlands, which were related to the difficulty of nutrient acquisition and soil acidity. Functional group predictions also indicated that the decrease in soil carbon and nitrogen contents was closely linked to the decrease in the functional groups of carbon and nitrogen transformations, which was determined by the changes in prokaryotic community composition. Indeed, short-term abandonment of ancient rice terraces does not facilitate the accumulation of soil carbon and nitrogen contents and leads to terrible ecological consequences. From the perspective of short-term abandonment, an ecological impact of the prevalence of the abandonment of rice terraces is most likely to be a reduction in soil carbon and nitrogen contents and their turnover in the hilly areas of China, which in turn leads to other ecological consequences.

## Conclusions

This study clarified that short-term abandonment of ancient rice terraces was not conducive to the accumulation of soil carbon and nitrogen. After ancient rice terraces were converted to drylands and forestlands, SOC (51.09 and 31.76%) and TN (33.20 and 16.59%) contents reduced significantly. The prevalence of the abandonment of ancient rice terraces may bring other adverse ecological consequences in the hilly areas of China. From the perspective of prokaryotic communities, it was shown that the abandonment of terraces reduced diversity and changed community composition and was mainly affected by pH and ${\text{NH}}_{4}^{+}$-N. Functional prediction also showed that the functional groups in carbon and nitrogen transformations decreased significantly after the abandonment of terraces and had a significant positive correlation with soil carbon and nitrogen contents. We conclude that the decrease of soil carbon and nitrogen contents after short-term abandonment of ancient rice terraces may be related to the decline of carbon and nitrogen-related prokaryotic functional groups. Therefore, future studies on the transformation and stability of soil elements should pay more attention to the gene changes of related microbial functional group. Notably, the ecological consequences of abandoning terrace in hilly areas also need to be noted, which may have adverse effects, such as slowing down the elemental cycle and exacerbating the greenhouse effect.

## Data availability statement

The sequencing data have been deposited in the National Center for Biotechnology Information (NCBI) Sequence Read Archive (SRA) database, and the bacterial and archaeal accession numbers were PRJNA895535 and PRJNA895578, respectively.

## Author contributions

WL: roles/writing—original draft, conceptualization, visualization, and writing—review and editing. ZL and YL: methodology. XN, ZL, and YL: validation and supervision. CD, ZL, and YL: funding acquisition. GZ, SW, TX, and HZ: investigation. All authors have read and agreed to the published version of the manuscript.

## Funding

This work was supported by the National Natural Science Foundation of China (U19A2047 and 41877084), under grants ZL and YL, respectively, to professors at School of Geographic Sciences, Hunan Normal University.

## Conflict of interest

The authors declare that the research was conducted in the absence of any commercial or financial relationships that could be construed as a potential conflict of interest.

## Publisher's note

All claims expressed in this article are solely those of the authors and do not necessarily represent those of their affiliated organizations, or those of the publisher, the editors and the reviewers. Any product that may be evaluated in this article, or claim that may be made by its manufacturer, is not guaranteed or endorsed by the publisher.
