# Supplementary material for: Reshaping of soil carbon and nitrogen contents in quincentenary ancient rice terraces: The role of both short-term abandonment and prokaryotic functional groups
Source: Front Microbiol. 2022 Dec 1;13:1007237. doi: 10.3389/fmicb.2022.1007237 (PMC9754093; doi:10.3389/fmicb.2022.1007237)
Supplement: Supplementary file 1 [file Data_Sheet_1.docx]

**
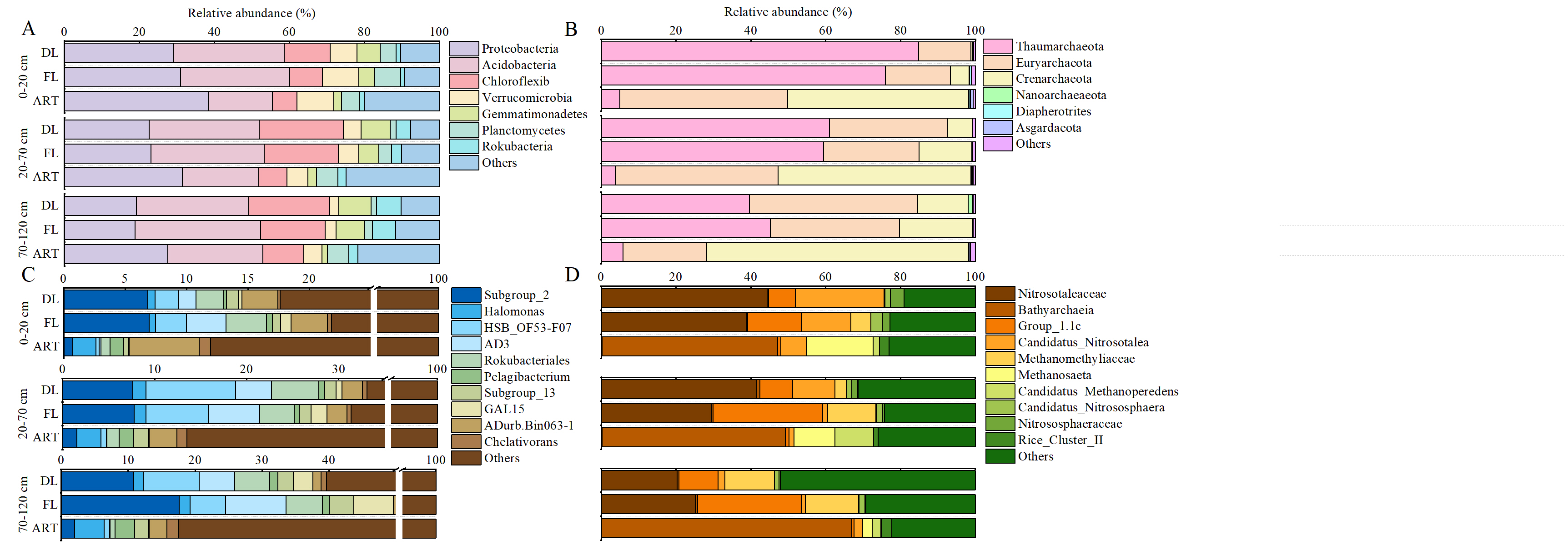
Fig. S1** The relative abundance (%) of dominant phyla and genera of soil prokaryotic communities of three land use types in different soil horizons. A and B are the phylum level of bacterial and archaeal communities; C and D are the genus level of bacterial and archaeal communities. DL: dry land; FL: forest land; ART: ancient rice terraces.


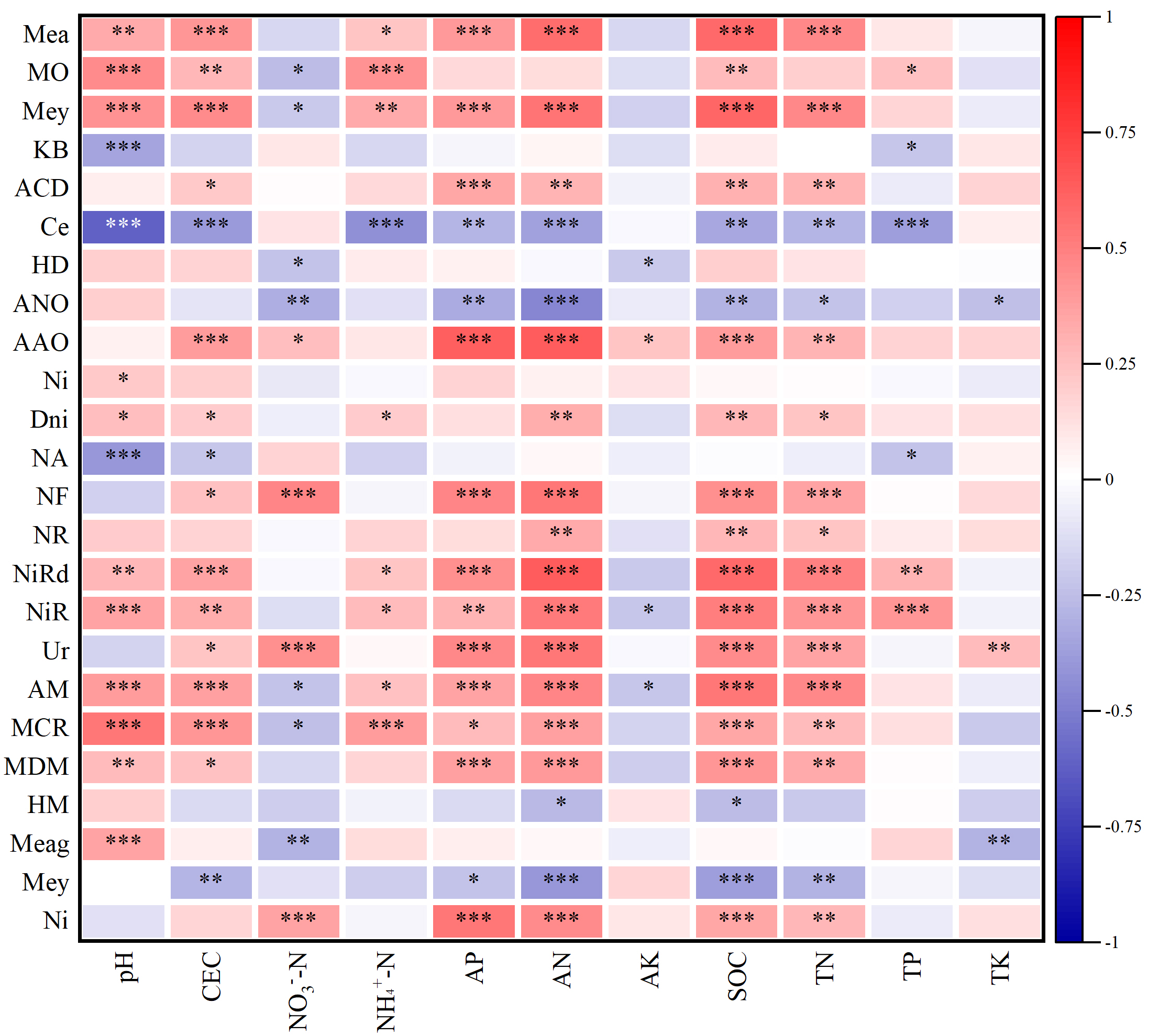
**Fig. S2** Correlation coefficient between prokaryotic carbon and nitrogen transformation functional groups and soil properties. Bacterial community: Mea: Methanotrophy; MO: Methanol oxidation; Mey: Methylotrophy; KB: Knallgas bacteria; Ce: Cellulolysis; ACD: Aromatic compound degradation; HD: Hydrocarbon degradation; AAO: aerobic ammonia oxidation; ANO: aerobic nitrite oxidation; Ni: nitrification; Dni: denitrification; NF: nitrogen fixation; NA: nitrate ammonification; NR: nitrite respiration; NiR: nitrate respiration; NiRd: nitrate reduction; Ur: ureolysis. Archaeal community: AM: acetoclastic_methanogenesis; MDM: methanogenesis by disproportionation of methyl groups; MCR: methanogenesis by CO_2_ reduction with H_2_; HM: hydrogenotrophic methanogenesis; Meag: methanogenesis; Mey: methylotrophy; Ni: nitrification. ^*^ *P* < 0.05; ^**^ *P* < 0.01; ^***^ *P* < 0.001.

**
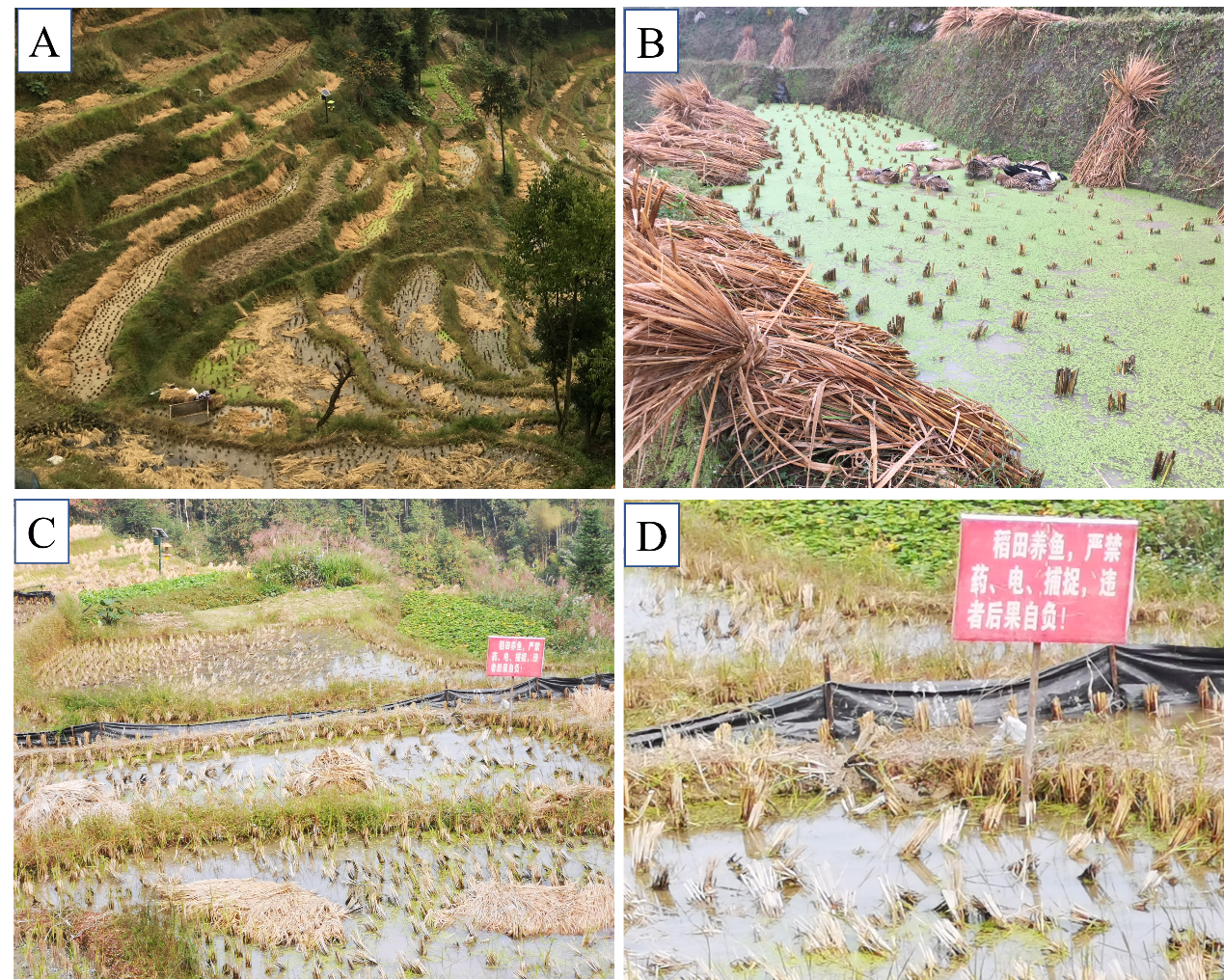
Fig. S3** More sources of soil nutrients for ancient rice terraces. A: straw returned to rice terraces; B: free-range ducks in rice terraces; C: farm fish in rice terraces; D: warning signs for farmed fish in rice terraces.

**Table S1** Radiocarbon dating results of soil samples from Ziquejie ancient rice terraces.

| Sample number | Soil depth | Percent modern carbon | ^14^C age | Calibrated age | Probability |
| --- | --- | --- | --- | --- | --- |
|  | cm | % | year BP | year | % |
| Beta–595583 | 0–20 | 94.43±0.35 | 460±30 | 1412–1471 AD | 95.4 |
| Beta–595584 | 60–80 | 89.40±0.33 | 900±30 | 1116–1219 AD | 60.5 |
| Beta–595585 | 100–120 | 34.20±0.13 | 8620±30 | 7728–7583 BC | 95.4 |

**Table S2** Soil properties of three land use types in the different soil horizons.

| LUT | SH | pH | CEC | NO_3_^-^-N | NH_4_^+^-N | AN | AP | AK | TP | TK |
| --- | --- | --- | --- | --- | --- | --- | --- | --- | --- | --- |
|  |  |  | cmol kg^-1^ | mg kg^-1^ | mg kg^-1^ | mg kg^-1^ | mg kg^-1^ | mg kg^-1^ | g kg^-1^ | g kg^-1^ |
| DL | A | 4.80±0.07Bab | 7.66±0.24Ba | 6.21±0.55Aa | 4.52±0.36Ca | 42.24±2.40Ba | 9.03±0.34Aa | 64.80±4.74Aa | 0.16±0.01Aa | 1.85±0.03Aa |
| FL |  | 4.81±0.05Bb | 7.03±0.63Ba | 4.56±0.71Ba | 6.34±0.29Ba | 42.10±5.24Ba | 3.47±0.36Ba | 24.20±3.31Ba | 0.15±0.00Aa | 1.79±0.06Aa |
| ART |  | 5.29±0.07Aa | 9.45±0.26Aa | 2.62±0.17Ca | 8.38±0.61Aab | 61.69±2.67Aa | 7.65±0.80Aa | 29.20±3.17Bb | 0.16±0.01Aa | 1.55±0.18Aa |
| DL | B | 4.60±0.05Bb | 6.62±0.29Bb | 4.82±0.41Ab | 3.86±0.36Ca | 31.32±2.18ABb | 4.39±0.52Ab | 55.70±5.01Aa | 0.15±0.00ABab | 1.85±0.11Aa |
| FL |  | 4.65±0.05Bc | 6.97±0.50Ba | 2.16±0.30Bb | 5.55±0.24Ba | 24.91±2.39Bb | 0.78±0.06Bb | 15.50±1.83Bb | 0.14±0.00Bab | 1.58±0.14ABab |
| ART |  | 5.39±0.04Aa | 8.16±0.23Ab | 1.46±0.15Bb | 6.86±0.56Ab | 35.85±2.72Ab | 4.63±0.33Ab | 15.80±1.49Bc | 0.16±0.01Aa | 1.49±0.10Ba |
| DL | C | 4.97±0.10Ba | 6.85±0.18Bb | 3.37±0.25Ac | 4.02±0.46Ba | 14.85±1.88Bc | 2.21±0.46ABc | 61.53±4.12Aa | 0.15±0.00Bb | 1.70±0.09Aa |
| FL |  | 4.97±0.04Ba | 5.40±0.48Ca | 1.81±0.28Bb | 3.74±0.40Bb | 13.43±1.96Bc | 1.30±0.26Bb | 10.73±0.62Cb | 0.13±0.00Cb | 1.37±0.07Bb |
| ART |  | 5.42±0.07Aa | 7.80±0.23Ab | 1.88±0.12Bb | 10.87±1.10Aa | 26.24±1.69Ac | 3.14±0.28Ac | 39.73±2.17Ba | 0.17±0.01Aa | 1.23±0.10Ba |
| *Variance analysis of Two-way ANOVA* | | | | | | | | | | |
| LUT | | 50.19^***^ | 19.01^***^ | 55.95^***^ | 29.12^***^ | 25.73^***^ | 57.4^***^ | 124.46^***^ | 10.71^***^ | 7.81^**^ |
| SH | | 10.37^***^ | 8.00^**^ | 27.61^***^ | 1.52^ns^ | 97.13^***^ | 72.29^***^ | 7.90^**^ | 0.63^ns^ | 5.95^**^ |
| LUT×SH | | 2.00^ns^ | 2.00^ns^ | 4.76^**^ | 6.28^***^ | 1.80^ns^ | 8.00^***^ | 6.99^***^ | 1.40^ns^ | 0.37^ns^ |

DL: dry land; FL: forest land; ART: Ancient rice terraces; A: 0–20 cm; B: 20–70 cm; C: 70–120 cm; CEC: cation exchange capacity; NO_3_^–^–N nitrate nitrogen; NH_4_^+^–N: ammonium nitrogen; AN: alkaline nitrogen; AP: available phosphorus; AK: available potassium; TP: total phosphorus; TK: total potassium; LUT: land use types; SH: soil horizons. Various capital letters stand for significant differences among diverse land use types, diverse lower-case letters indicate significant differences among diverse soil horizons. ^*^ *P* < 0.05; ^**^ *P* < 0.01; ^***^ *P* < 0.001; ^ns^ not significant.

**Table S3** Basic information of soil prokaryotic community from high throughput sequencing of three land use types in the different soil horizons.

| LUT | SH | Bacterial community | | |  | Archaeal community | | |
| --- | --- | --- | --- | --- | --- | --- | --- | --- |
|  |  | Reads | |  |  | Reads | |  |
|  |  | BFS | AFS | Coverage (%) |  | BFS | AFS | Coverage (%) |
| DL | A | 88653±1785 | 63518 | 99.60±0.02 |  | 51191±2322 | 32547 | 99.46±0.07 |
| FL |  | 88886±1855 | 63518 | 99.70±0.04 |  | 54711±689 | 32547 | 99.66±0.05 |
| ART |  | 78828±4561 | 63518 | 99.66±0.06 |  | 44487±1597 | 32547 | 98.71±0.26 |
| DL | B | 92525±2010 | 63518 | 99.72±0.03 |  | 51421±1658 | 32547 | 99.23±0.11 |
| FL |  | 90086±1418 | 63518 | 99.73±0.03 |  | 55303±1735 | 32547 | 98.32±0.29 |
| ART |  | 93210±5015 | 63518 | 99.74±0.04 |  | 44448±1716 | 32547 | 98.02±0.31 |
| DL | C | 96455±2011 | 63518 | 99.82±0.02 |  | 54816±1968 | 32547 | 99.24±0.13 |
| FL |  | 94233±2893 | 63518 | 99.86±0.03 |  | 56482±1544 | 32547 | 98.83±0.19 |
| ART |  | 98154±3552 | 63518 | 99.77±0.02 |  | 47518±2926 | 32547 | 98.12±0.21 |

DL: Dry land; FL: Forest land; ART: Ancient rice terraces; A: 0–20 cm; B: 20–70 cm; C: 70–120 cm; LUT: land use types; SH: soil horizons; BFS: before filtering and standardization; AFS: after filtering and standardization.
